# Texture Analysis and Radial Basis Function Approximation for IVUS Image Segmentation

**DOI:** 10.2174/1874120700701010053

**Published:** 2007-09-28

**Authors:** Maria Papadogiorgaki, Vasileios Mezaris, Yiannis S Chatzizisis, George D Giannoglou, Ioannis Kompatsiaris

**Affiliations:** 1 Informatics and Telematics Institute, Centre for Research and Technology Hellas, 1st Km Thermi-Panorama Rd, P.O. Box 60361, GR-57001 Thermi-Thessaloniki, Greece; 2 Cardiovascular Engineering and Atherosclerosis Laboratory, AHEPA University Hospital, Aristotle University Medical School, GR-54636, Thessaloniki, Greece

**Keywords:** Intravascular ultrasound, coronary arteries, image segmentation, lumen, media, adventitia, boundaries, texture analysis.

## Abstract

>Intravascular ultrasound (IVUS) has become in the last years an important tool in both clinical and research applications. The detection of lumen and media-adventitia borders in IVUS images represents a first necessary step in the utilization of the IVUS data for the 3D reconstruction of human coronary arteries and the reliable quantitative assessment of the atherosclerotic lesions. To serve this goal, a fully automated technique for the detection of lumen and media-adventitia boundaries has been developed. This comprises two different steps for contour initialization, one for each corresponding contour of interest, based on the results of texture analysis, and a procedure for approximating the initialization results with smooth continuous curves. A multilevel Discrete Wavelet Frames decomposition is used for texture analysis, whereas Radial Basis Function approximation is employed for producing smooth contours. The proposed method shows promising results compared to a previous approach for texture-based IVUS image analysis.

## INTRODUCTION

Coronary angiography is acknowledged as the gold standard for imaging and diagnosis of coronary heart disease. However it is restricted by its inability to depict the vessel wall, provided that it illustrates the coronary arteries as a silhouette of the lumen. Thus, it fails to quantify plaque burden, responsible for partial or total obstruction of the arteries. Recently, intravascular ultrasound (IVUS) has been introduced as a complementary to angiography diagnostic technique aiming to more accurate imaging of coronary atherosclerosis [1, 2].

**Fig. (1):**
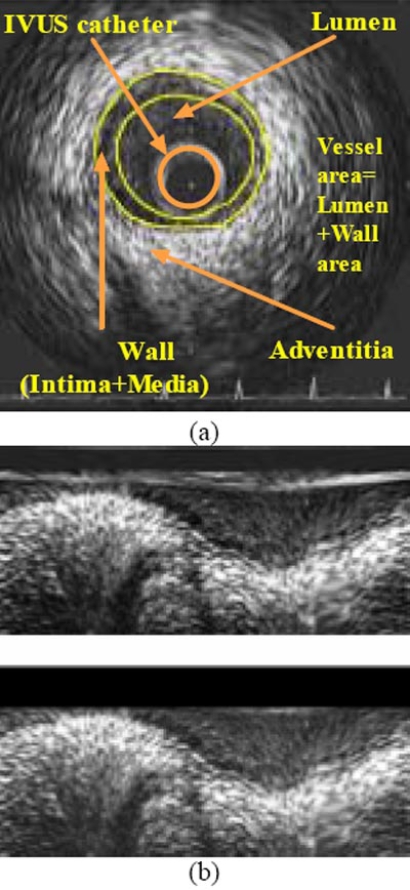
Original IVUS image (**a**) and corresponding polar coordinate images before and after (**b**) the removal of catheter-induced artifacts.

IVUS is a catheter-based technique that renders two-dimensional images of coronary arteries and therefore, provides information concerning luminal and wall area, plaque morphology and wall composition (Fig. **1**). However, the quantitative evaluation of characteristic parameters such as luminal and wall area that is necessary for the clinical evaluation of the image data requires their segmentation according to the actual structure of the coronary arteries. The wall of the latter mainly consists of three layers: intima, media and adventitia, while three regions are supposed to be visualized as distinguished fields in an IVUS image, namely the lumen, the vessel wall (consisted by the intima and the media layers) and the adventitia plus surroundings, as illustrated in (Fig. **1**). The above regions are separated by two closed contours: the inner border, which corresponds to the lumen-wall interface, and the outer border representing the boundary between media and adventitia. The reliable and quick detection of these two borders is the goal of analysis and also the basic step towards the subsequent 3D reconstruction of the arteries, which can provide additional information regarding the burden of atherosclerosis [3].

This study presents an automated method for the segmentation of IVUS images and specifically for the detection of luminal and medial-adventitial boundaries, based on the results of texture analysis, performed by means of a multilevel Discreet Wavelet Frames decomposition, and on a smoothing step using Radial Basis Function (RBF) approximation. The proposed approach does not require manual initialization of the contours and shows promising results.

## RELATED WORK

Traditionally, the segmentation of IVUS images is performed manually, which is a time consuming procedure with results affected by the high inter- and intra-user's variability. To overcome these limitations, several approaches for semi-automated segmentation have been proposed in the literature. Sonka *et al.* implemented a knowledge-based graph searching method incorporating a priori knowledge on coronary artery anatomy and a selected region of interest prior to the automatic border detection [4]. Quite a few variations of active contour model have been investigated. The active contour principles have been used to allow the extraction of the borders in three dimensions after setting an initial contour in Kovalski *et al's.* approach [5]. However, the contour detection fails for low contrast interface regions such as the luminal border where the blood-wall interface in most images corresponds to weak pixel intensity variation. Klingensmith *et al. *use the frequency information to improve the active surface segmentation algorithms after acquiring the radiofrequency (RF) IVUS data, for plaque characterization [6].

For clinical practice the most attractive approaches are the fully automatic ones. A limited number of them has been developed so far. Brusseau *et al.* [7] exploited an automatic method for detecting the endoluminal border based on an active contour that evolves until it optimally separates regions with different statistical properties. A fuzzy clustering algorithm for adaptive segmentation in IVUS images [8] is investigated by Filho *et al*. Giannoglou *et al. *propose in [9] an automated segmentation method based on a variant of the active contour model. Cardinal *et al.* present a 3D IVUS segmentation where Rayleigh probability density functions (PDFs) are applied for modeling the pixel gray value distribution of the vessel wall structures [10]. An automated approach based on deformable models has been reported by Plissiti *et al. *[11], who employed a Hopfield neural network for the modification and minimization of an energy function as well as a priori vessel geometry knowledge. Unal *et al. *proposed in [12] a shape-driven approach to the segmentation of IVUS images, based on building a shape space using training data and consequently constraining the lumen and media-adventitia contours to a smooth, closed geometry in this space.

## IVUS IMAGE PREPROCESSING

Preprocessing of the image data for the purpose of applying a texture description method consists of two steps: (a) representation of the images in polar coordinates, and (b) removal of catheter-induced artifacts.

Representation of the images in polar coordinates is important for facilitating the description of local image regions in terms of their radial and tangential characteristics. It also facilitates a number of other detections steps, such as contour initialization and the smoothing of the obtained contour. To this end, each of the original IVUS images is transformed to a polar coordinate image where columns and rows correspond to angle and distance from the center of the catheter, respectively, and this image alone, denoted *I* (*r,θ*), is used throughout the analysis process.

The images produced by IVUS include not only tissue and blood regions but also the outer boundary of the catheter itself. The latter defines a dead zone of radius equal to that of the catheter, where no useful information is contained. Knowing the diameter *D* of the catheter, these catheter-induced artifacts are easily removed by setting *I* (*r,θ*) = 0 for *r < D / 2 + e*, *e* being a small constant. For the purpose of experimentation ***e*** was set to 4; any other positive value that would be small enough so as not to result in the removal of parts of the wall area would suffice. This preprocessing is illustrated in Fig. (**1**).

## TEXTURE ANALYSIS

Texture has been shown to be an important cue for the analysis of generic images [13]. In this work, the Discrete Wavelet Frames (DWF) decomposition [14] is used for detecting and characterizing texture properties in the neighborhood of each pixel. This is a method similar to the Discrete Wavelet Transform (DWT) that uses a filter bank to decompose the grayscale image to a set of subbands. The main difference between DWT and DWF is that in the latter the output of the filter bank is not subsampled. The DWF approach has been proven to decrease the variability of the estimated texture features, thus improving pixel classification for the purpose of image segmentation. The employed filter bank is based on the lowpass Haar filter:

(1) $$H\left(z\right)=\frac{1}{2}\left(1+z^{-1}\right)$$

Using this along with the complementary highpass filter *G*(*z*), defined as $G\left(z\right)=\mathrm{zH}\left({-z}^{-1}\right)$ , the fast iterative scheme proposed in [14] for applying the DWF analysis in the two-dimensional space is realized. Then, according to the DWF theory, the texture of pixel *p* can be characterized by the standard deviations of all detail components, calculated in a neighborhood *F* of pixel *p*. The images resulting from treating each calculated standard deviation as intensity value of pixel *p* are denoted as *I_k,_* *k* = 1,...,*K*. In the proposed approach, a DWF decomposition of four levels is employed, resulting in *K* = 12 such images, in addition to an approximation component, which is a low-pass filtered image denoted *I_LL_*. However, not all of these images are used for the localization of the contours, as discussed in the sequel.

## CONTOUR INITIALIZATION

Objective of the contour initialization procedure is the detection of pixels that are likely to belong to the lumen and media-adventitia boundaries.

Under the proposed approach, the initialization of the lumen boundary relies in the observation that the lumen and wall areas demonstrate different texture characteristics: the lumen area tends to be a low-intensity non-textured region, with noise being responsible for any high-intensity artifacts in it, whereas the wall area is typically characterized by the presence of both low-intensity and high-intensity parts, with changes between the two that are of relatively low-frequency in the tangential direction and of somewhat higher frequency in the radial direction. Consequently, the local energy of the signal in appropriate frequency sub-bands can be used as a criterion for differentiating between the lumen and wall areas; to this end, the results of texture analysis discussed in section Texture Analysis are employed.

For the initialization of the media-adventitia boundary, on the other hand, the approximation component of the DWF decomposition is employed. The motivation behind this choice lies in the observation that in many cases the latter is represented by a thick bright ring (a thick bright curve in polar coordinates) that is dominant in the image. Thus, in the approximation component, the media-adventitia boundary is rather well preserved, as opposed to higher-frequency details that are suppressed by the low-pass filtering, facilitating contour initialization.

More specifically, let *I*_int_, *I_ext_* denote the simplified images, after texture analysis, which are used for detecting the lumen and media-adventitia boundaries respectively. These are defined under the proposed approach as

(2) $$\begin{array}{cc} I_{\mathrm{int}} & \left(r,\theta \right)=\frac{255}{\max_{\left(r,\theta \right)}\left\{I_{\mathrm{int}}^{'}\left(r,\theta \right)\right\}}I_{\mathrm{int}}^{'} \end{array}\left(r,\theta \right)$$

(3) $$I_{\text{int}}^{'}\left(r,\theta \right)=\sum_{k}{\text{I}}_{k}\left(r,\theta \right)$$

(4) $$I_{\mathrm{ext}}\left(r,\theta \right)=I_{\mathrm{LL}}\left(r,\theta \right)$$

The choice of the images I_*k*_ that are employed in this initialization process was done based on visual evaluation of all *K* generated images and is in line with the aforementioned observations regarding the texture properties of the lumen and wall areas, in combination with the characteristics of the filter bank used for the generation of images I_*k*_

The internal contour is initialized as the set of pixels

(5) $$c_{\text{int}}=\left\{p_{\text{int}}=\left[\rho ,\theta \right]\right\}$$

for which

(6) $${\text{I}}_{\text{int}}\left(\rho ,\theta \right)>T\text{ and }{\text{I}}_{\text{int}}\left(r,\theta \right)<\text{T}\forall r<\rho$$

thus defining an internal contour function *C*_int_ (*θ*) = *ρ* (Fig. (**2a**)). *T* in the above equation is a threshold whose value was set experimentally to 128; small deviations from this value where shown to have little effect on the results of initialization.

The external contour is initialized as the set of pixels:

(7) $$c_{\mathrm{ext}}=\left\{p_{\mathrm{ext}}=\left[\mu ,\theta \right]\right\}$$

for which

(8) $${\text{I}}_{\mathrm{ext}}\left(\mu ,\theta \right)=\underset{r>\rho^{'}}{\text{max}}\left\{I_{\mathrm{ext}}\left(r,\theta \right)\right\}$$

where [*ρ',θ*] are the points of the final internal contour, as obtained by applying to the initialization data the refinement process of the following section. This defines a contour function *C_ext_* (*θ*) = *μ* for the external contour (Fig. (**2b**)).

## RBF-BASED CONTOUR REFINEMENT

**Fig. (2):**
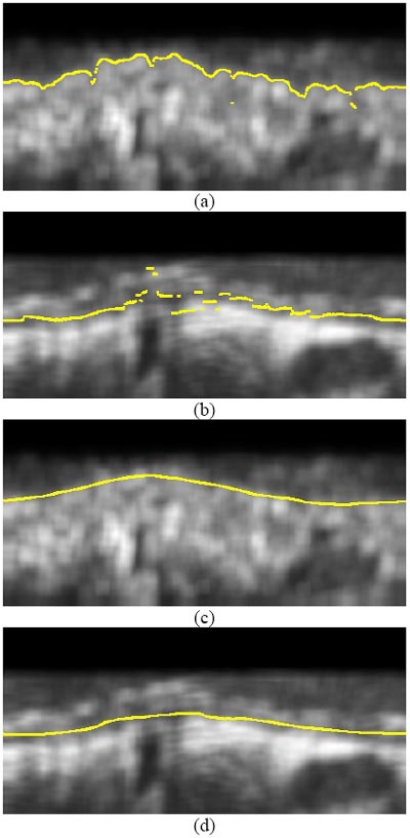
Results of contour initialization for (**a**) the lumen, (**b**) the media-adventitia boundary, and (**c**), (**d**) the corresponding contours after contour refinement.

In contrast to the initial contours generated as described in the previous section, which are not smooth and are characterized by discontinuities (Fig. **2**), the true lumen and media-adventitia boundaries are smooth, continuous functions of *θ*. Consequently, in order to obtain smooth contours that are consistent with the true ones, the application of a filtering or approximation procedure to the initial contour functions *C*_int_ (*θ*), *C_ext_* (*θ*) is required. In this work, RBFs [15] are used to this end.

Polyharmonic RBFs have been proposed for reconstructing smooth, manifold surfaces from point-cloud data and for repairing incomplete meshes through interpolation methods and approximation techniques. Their use for the approximation of the initial contours in a frame, i.e. the generation of a contour *c'* that is a smooth, reasonable approximation of *c* requires the definition for each such contour of a function *f* , as follows:

(9) $$f\left(\theta ,C\left(\theta \right)\right)=0$$

where *C*(*θ*) here denotes either *C*_int_(*θ*) or *C_ext_*(*θ*), depending on the contour being examined. Function *f* is used for formulating the approximation problem as one of finding an RBF *s* for which $s\left(.\right)≅f\left(.\right).$ To avoid the trivial solution of ***s*** being zero at every point, *f* must also be defined for a set of points not belonging to the initial contour, so that

(10) $$f\left(\theta ,r\neq C\left(\theta \right)\right)\neq 0$$

The latter points are defined in this work as those which satisfy the following equations:

(11) $$r=\max_{\theta }\left\{C\left(\theta \right)\right\}+1$$

(12) $$r=\max_{\theta }\left\{C\left(\theta \right)\right\}-1$$

For the above points in the 2D space, function *f* is defined as the signed Euclidean distance from the initialized contour for *θ* = *const* , i.e.

(13) $$f\left(\theta ,r\neq C\left(\theta \right)\right)=r-C\left(\theta \right)$$

Following the definition of *f*, the FastRBF library [16] was used to generate the smooth contour approximation *c'* by removing duplicate points where *f* has been defined (i.e. points in the 2D space which are located within a specific minimum distance from other input points), fitting of an RBF to this data and evaluating it to find the points which correspond to zero value; the latter define the contour approximation *c'*.

## EXPERIMENTAL RESULTS

The developed IVUS image analysis methodology was applied to a set of 40 images randomly selected from a pool of approximately 300 images belonging to 5 different human arterial segments; the 40 randomly selected images were segmented manually by experts to generate ground truth results. Similarly to [17], the arterial segments were captured using a mechanical imaging system and a 2.6F sheath-based catheter, incorporating a 40 MHz single-element transducer rotating at 1800 rpm and generating 30 images/sec. A motorized pullback device was used to draw out the catheter at a constant speed of 0.5 mm/sec. The ultrasound data was recorded in a 0.5-inch S-VHS videotape. The S-VHS data was digitized by an integrated to the IVUS console frame grabber at 512x512 pixels with 8-bit grey scale in a rate of 7.5 images/sec and the end-diastolic images were selected (peak of R-wave on ECG).

**Fig. (3):**
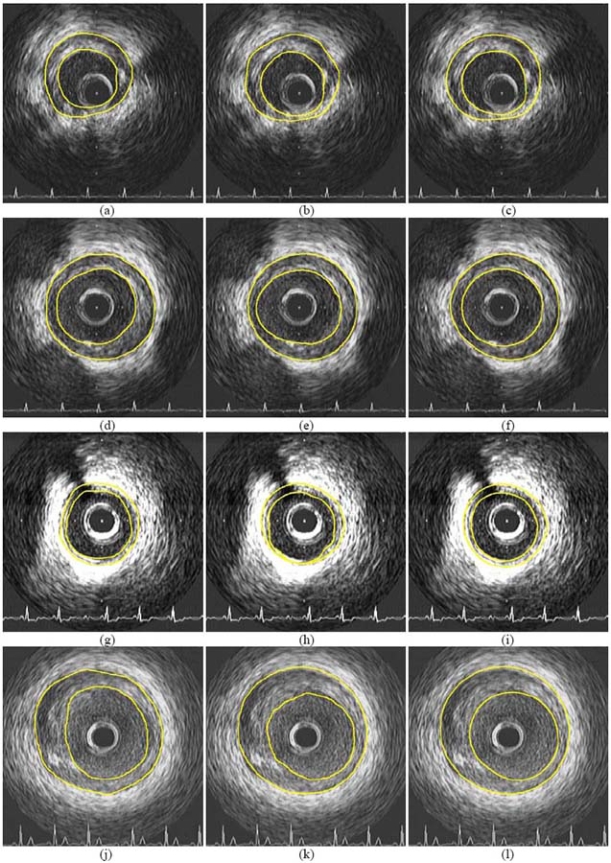
Indicative experimental results of the proposed approach (right column) and comparison with the results of [17] (middle column) and corresponding contours manually generated by experts (left column).

**Fig. (4):**
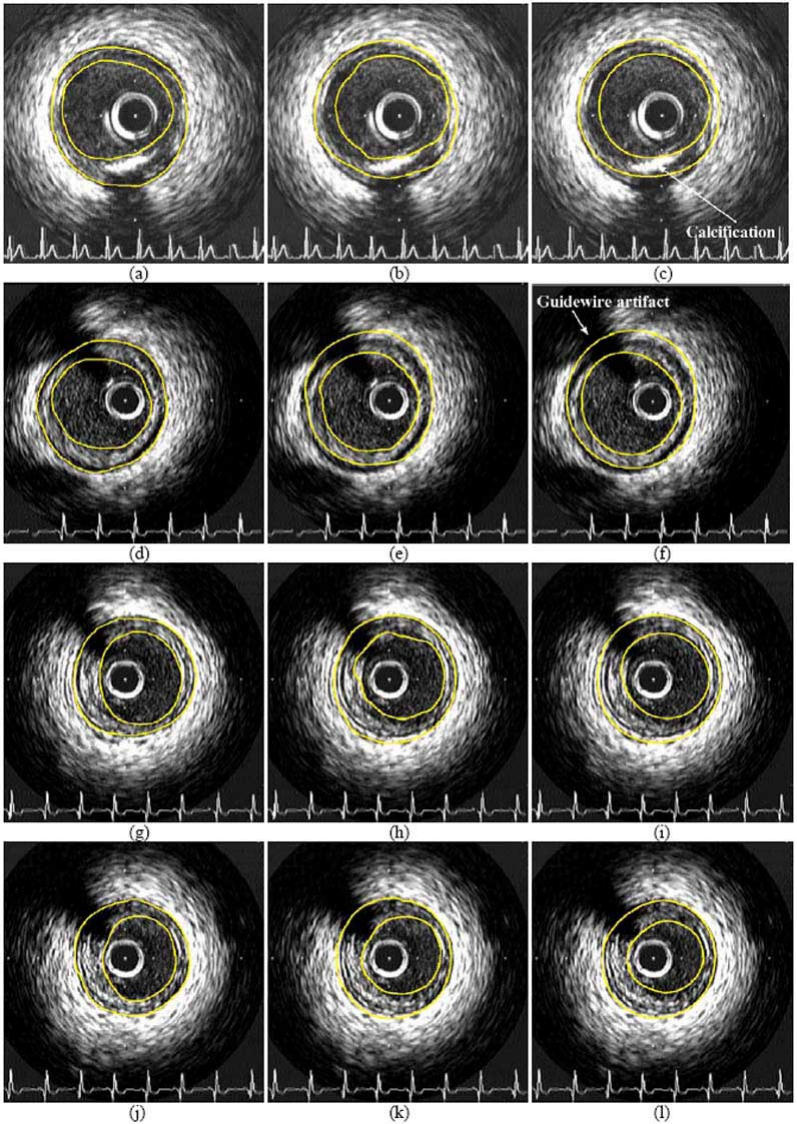
Indicative experimental results of the proposed approach (right column) and comparison with the results of [17] (middle column) and corresponding contours manually generated by experts (left column), including images with calcification and guidewire artifacts.

Indicative results of the proposed approach on the employed dataset are presented in (Figs. **3** and **4**), where the boundaries manually detected by a domain expert are also shown, along with results of our previous approach. Numerical comparison of the aforementioned methods, using parameter values that experts have defined and use for evaluating the results of IVUS (regardless of whether segmentation is performed manually or otherwise) and ground truth results, is shown in Table **1**.

## DISCUSSION

The evaluation of the experimental results presented in the previous section documents the improved performance of the proposed approach as compared to our previous one in [17]. This improvement is due to the introduction of an elaborate contour refinement process based on RBFs for processing the results of contour initialization.

The aforementioned results also demonstrate the good agreement between the output of the proposed automated approach and that of manual segmentation, rendering promising the future exploitation of it in clinical practice.

## CONCLUSIONS

In this paper an automated approach for the detection of lumen and media-adventitia boundaries in IVUS images has been presented, based on the results of texture analysis and the use of RBFs. The experiments conducted by applying the developed technique to real data showed promising results and demonstrated the usefulness of this technique for IVUS image analysis. Future developments of this work include the combination of the texture features with other features reported in the related literature for contour initialization and the extensive evaluation of the technique.

## Figures and Tables

**Table 1.** Average Error (%) for Vessel Area (VCSA), Lumen Area (LCSA) and Wall Area (WCSA) (see Fig. 1) Calculated from the Segmentation Results, Using the Method of [17] and the Proposed One. Ground Truth Values for VCSA, LCSA and WCSA have been Calculated Using the Results of Manual Segmentation by Experts

Parameter/Method	Method of [17]	Proposed Approach
**Vessel area (VCSA)**	7.51%	5.57%
**Lumen area (LCSA)**	11.09%	8.95%
**Wall area (WCSA)**	17.34%	10.81%
